# Bilateral squamous cell carcinoma in horse shoe kidney

**DOI:** 10.1016/j.eucr.2024.102695

**Published:** 2024-02-29

**Authors:** Abbas Basiri, Mehdi dadpour, Sobhan Sabzi, Peyman Mohammadi Torbati, Fereshteh Kimia

**Affiliations:** a Urology and Nephrology Research Center, Shahid Labbafinejad Medical Center, Shahid Beheshti University of Medical Sciences, Tehran, Iran; b Anesthesiology Department, Labbafinejad Hospital, Shahid Beheshti University of Medical Scieces, Tehran, Iran

**Keywords:** Squamous cell carcinoma, Horseshoe kidney, Kidney anomaly, Kidney neoplasm

## Abstract

To present a patient with horseshoe kidney and bilateral squamous cell carcinoma (SCC) which has not been reported so far. A 61-year-old woman presented with abdominal mass and recent episodes of gross hematuria. Imaging revealed malignant lesion of lower calyces of the right kidney and isthmus of horse-shoe kidney with midline crossing to the left side. Finally, the patient underwent bilateral enbloc radical nephroureterectomy and pathology evaluation was compatible with bilateral squamous cell carcinoma. This is the first report of bilateral SCC in horseshoe kidney which was managed via open enbloc radical nephroureterectomy.

## 1 Introduction

Horseshoe kidney as a congenital anomaly in which the two kidneys are attached to each other at their lower poles approximately occurs in 1 per 400 individuals and it is twice as common in men. Kidney stones, partial uretero-pelvic obstruction, vesico-ureteral reflux and kidney tumors are more likely to occur in this anomaly.^1^^,^^2^ Both open and minimally invasive surgical management of horseshoe kidney tumors are interesting issues in the literature. Upper Tract Urothelial Carcinoma (UTUC) is accounting for more than 90% of renal pelvis tumors and 5%–7% of all kidney tumors which is considered as an uncommon diagnosis, while squamous cell carcinoma (SCC) is accounting less than 7% of renal pelvic tumors which is still a rare finding.^3^, ^4^, ^5^ Radical Nephroureteretomy (RNU) is the gold standard treatment modality for UTUC, but if feasible, nephron sparing surgery is preferred for low grade tumors specially in bilateral involvement, single kidney and chronic kidney diseases. The role of neoadjuvant chemotherapy in UTUC has not been widely adopted as standard treatment.^6^

There are some limited reports of unilateral UTUC^7^ and SCC^8^^,^^9^ in horseshoe kidney and its different managements in the literature. In this report, we present a patient with horseshoe kidney, who was treated with the initial diagnosis of bilateral UTUC, and the final diagnosis of bilateral horseshoe kidney SCC was made in surgical pathology, which according to our knowledge has not been reported so far.

## 2 Case report

**Fig. 1:**
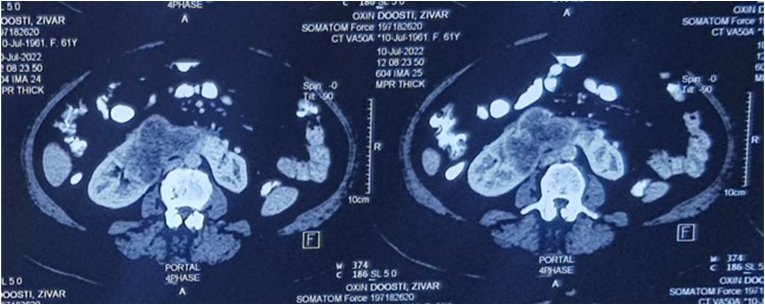
Abdomino-pelvic CT scan revealed malignant lesion originated from lower calyces of the right kidney and isthmus of horse-shoe kidney with 15 mm midline crossing to the left side of the isthmus.

**Fig. 2:**
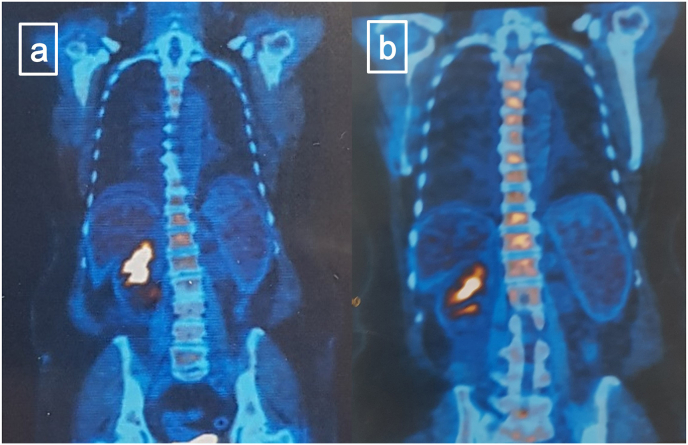
(a) Horse-shoe kidney with large hypermetabolic tumoral mass and no evidence of distant hypermetabolic metastatic lesions was reported in pre-chemotherapy PET-CT scan. (b) PET-CT scan following neoadjuvant chemotherapy revealed no evidence of distant hypermetabolic metastatic lesions and minimal decrease in size and metabolic activity of the malignant lesion comparing to the previous scan.

A 61-year-old woman presented with old abdominal pain and discomfort and some recent episodes of gross hematuria since 2 days before presentation. In primary evaluation she complained tenderness during deep palpation of her abdomen. Except for the pale conjunctiva, almost all the rest of physical examination were unremarkable. In the laboratory tests she had hemoglobin 8.5 g/dl and many RBCs in her urine analysis. The other parameters including GFR (85 ml/min/1.73m^2^), electrolytes and acute-phase reactants were within the normal range. Abdomino-pelvic CT scan revealed malignant lesion originated from lower calyces of the right kidney and isthmus of horse-shoe kidney with 15 mm midline crossing to the left side of the isthmus which was suspicious for upper urinary tract urothelial carcinoma (UTUC) (Fig. 1). Considering the rarity of bilateral UTUC in horse-shoe kidney and since if confirmed, bilateral RNU and long-life dialysis is the definitive treatment, the patient became candidate for further assessment. In the cysto-ureteroscopy evaluation there was no abnormal findings in the bladder but tumoral lesions were detected in pelvis and lower calyces of the right kidney and lower calyces of the left kidney which appeared to be derived from isthmus. Urine cytology showed atypical cells in the right kidney but it was negative for malignancy in the left kidney. Pathologic evaluation of cold-cup biopsy revealed high grade invasive urothelial carcinoma. Horse-shoe kidney with large hypermetabolic tumoral mass and no evidence of distant hypermetabolic metastatic lesions was reported in PET-CT scan (Fig. 2a).

**Fig. 3:**
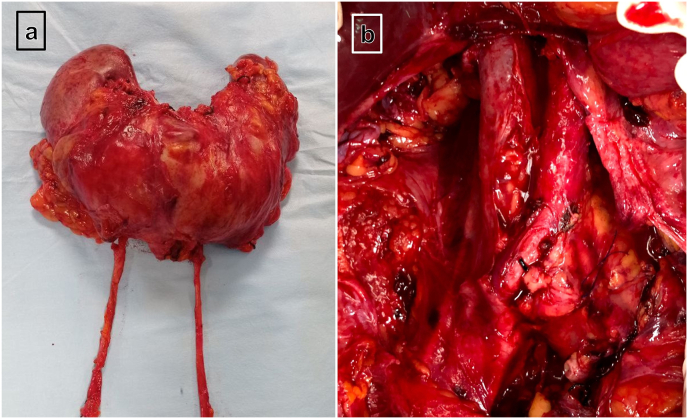
(a) bilateral enbloc radical nephroureterectomy. (b) Aorta and IVC are shown after specimen removal.

**Fig. 4:**
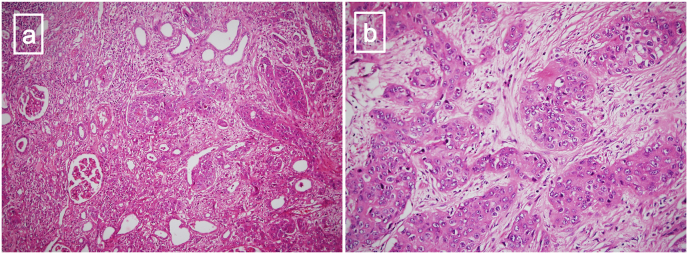
(a) Well differentiated squamous cell carcinoma of the renal pelvic extended into cortical parenchyma (H&E, 40*) (b) Pleomorphic squamous cells arranged in infiltrating sheets and nests surrounded by desmoplastic stroma (H&E, 400*).

Considering all above, bilateral RNU and long life dialysis as the gold standard treatment of bilateral UTUC was recommended to patient but despite the great emphasis, unfortunately she refused. The patient became candidate for neoadjuvant chemotherapy and if possible, partial nephrectomy as the second but not standard option. PET-CT scan following neoadjuvant chemotherapy revealed no evidence of distant hypermetabolic metastatic lesions and minimal decrease in size and metabolic activity of the malignant lesion comparing to the previous scan (Fig. 2b). To confirm this finding and evaluate the possibility of partial nephrectomy, the patient underwent cystoureteroscopy for the second time and unfortunately, bilateral local expansion of the tumor was observed directly. Finally, bilateral radical nephroureterectomy and regional lymphadenectomy as the only available treatment was performed after consultation with the patient (Fig. 3). Pathology evaluation revealed Pleomorphic squamous cells arranged in infiltrating sheets and nests surrounded by desmoplastic stroma which was compatible with Well differentiated squamous cell carcinoma of the renal pelvic extended into cortical parenchyma (Fig. 4). After 12 months follow up, no local recurrence or distant metastasis were detected.

## 3 Discussion

Horseshoe kidney and upper tract SCC are both unusual findings, and co-occurrence of these two conditions is scarce. This situation (unilateral tumor) was managed through open, laparoscopic or robotic radical nephrectomy (RN) in previous reports. Here we reported a patient with horseshoe kidney and bilateral SCC which to the best of our knowledge is the first report in the literature. The most important risk factor for renal squamous cell carcinoma is nephrolithiasis. Recurrent infection, smoking, high blood pressure, family history and advanced kidney disease could also be related to renal SCC.^10^ However, this patient did not have any mentioned risk factor.

Bilateral RN and long-life dialysis as the standard treatment can make the lifestyle very difficult. This is why patients hardly accept this kind of treatment. Mahesh et al.^11^ reported 12 patients who underwent partial nephrectomy (PN) for UTUC. The overall long term survival rate was 50% in their study. Considering the patient refusal for bilateral RNU in this report, PN appeared to be an alternative option if possible. Considering that UTUC was the primary diagnosis in our mind,

we suggested the patient neoadjuvant chemotherapy with the possibility of survival outcomes improvement and pathologic downstaging. In a systematic review, Oswald et al.^12^ concluded that neoadjuvant chemotherapy before RNU is associated with pathologic downstaging and have some oncological benefits in terms of overall survival and progression free survival. In our case, in spite of neoadjuvant chemotherapy, the patient experienced mass size increasing and tumor extension to the renal pelvis and as a result PN became impossible. However, these findings were not consistent with those of the follow up PET scan. Generally, this finding may be because of ineffective chemotherapy or pathologic misdiagnosis. In this case, the pathologist explanation about apparent discrepancy between the histologic type of the tumor in biopsy specimen and radical nephroureterectomy specimen was related to tumor heterogenicity and divergent squamous differentiation in different parts of the tumor. It does not seem that the urothelial carcinoma tumor would turn into squamous cell carcinoma tumor after chemotherapy, but eradicating portion of urothelial in the specimen and persistence of squamous cell carcinoma may be probable. Finally, bilateral RNU was performed as the best and only remaining option for the patient.

## 4 Conclusion

This is the first report of bilateral SCC in horseshoe kidney which was managed via open enbloc radical nephroureterectomy as the preferred treatment modality in this patient.

## CRediT authorship contribution statement

**Abbas Basiri:** Writing – review & editing, Supervision, Project administration, Data curation, Conceptualization. **Mehdi dadpour:** Writing – review & editing, Writing – original draft, Supervision, Data curation, Conceptualization. **Sobhan Sabzi:** Investigation, Data curation. **Peyman Mohammadi Torbati:** Supervision, Data curation. **Fereshteh Kimia:** Data curation.
